# The Astronomy of Africa’s Health Systems Literature During the MDG Era: Where Are the Systems Clusters?

**DOI:** 10.9745/GHSP-D-15-00034

**Published:** 2015-09-10

**Authors:** James F Phillips, Mallory Sheff, Christopher B Boyer

**Affiliations:** ^a^​ Columbia University, Mailman School of Public Health, Heilbrunn Department of Population and Family Health, New York, NY, USA

## Abstract

Growing international concern about the need for improved health systems in Africa has catalyzed an expansion of the health systems literature. This review applies a bibliometric procedure to analyze the acceleration of scientific writing on this theme. We focus on research published during the Millennium Development Goal (MDG) era between 1990 and 2014, reporting findings from a systematic review of a database comprised of 17,655 articles about health systems themes from sub-Saharan African countries or subregions. Using bibliometric tools for co-word textual analysis, we analyzed the incidence and associations of keywords and phrases to generate and visualize topical foci on health systems as clusters of themes, much in the manner that astronomers represent groupings of stars as galaxies of celestial entities. The association of keywords defines their relative position, with the size of images weighted by the relative frequency of terms. Sets of associated keywords are arrayed as stars that cluster as “galaxies” of concepts in the knowledge universe represented by health systems research from sub-Saharan Africa. Results show that health systems research is dominated by literature on diseases and categorical systems research topics, rather than on systems science that cuts across diseases or specific systemic themes. Systems research is highly developed in South Africa but relatively uncommon elsewhere in the region. “Black holes” are identified by searching for terms in our keyword library related to terms in widely cited reviews of health systems. Results identify several themes that are unexpectedly uncommon in the country-specific health systems literature. This includes research on the processes of achieving systems change, the health impact of systems strengthening, processes that explain the systems determinants of health outcomes, or systematic study of organizational dysfunction and ways to improve system performance. Research quantifying the relationship of governance indicators to health systems strengthening is nearly absent from the literature. Long-term experimental studies and statistically rigorous research on cross-cutting themes of health systems strengthening are rare. Studies of organizational malaise or corruption are virtually absent. Trend analysis shows the emergence of organizational research on specific priority diseases, such as on HIV/AIDS, malaria, and tuberculosis, but portrays a lack of focus on integrated systems research on the general burden of disease. If health systems in Africa are to be strengthened, then organizational change research must be a more concerted focus in the future than has been the case in the past.

## Abstract (precis)

The volume of literature on health systems in sub-Saharan Africa has been expanding since the 2000 MDG era. Focus has remained generally on categorical health themes rather than systems concepts. Topics such as scaling-up, organizational development, data use for decision making, logistics, and financial planning remain underrepresented. And quite surprisingly, implementation science remains something of a “black hole.” But bibliometric evidence suggests there is a shift in focus that may soon address these gaps.

## INTRODUCTION

The global literature on health systems policy, implementation, and research has proliferated in recent decades.^1^ In sub-Saharan Africa, investment in health systems development has been transformative, generating a wide array of scientific and policy articles on problems encountered and lessons learned.^2^^-^^6^

This commitment to health systems development in Africa can be traced to modality innovations, disease-control initiatives, and vertical programs launched in the 1970s and 1980s.^6^ The 1978 Alma Ata Conference catalyzed critically important health systems action and writing.^7^^-^^9^ The subsequent focus of the international community on health systems integration, reform, and decentralization received impetus from international partnerships. For example, the “sector-wide approach,” financed by the World Bank and other international initiatives,^10^ influenced policies and program implementation throughout the region.^11^^,^^12^ With the onset of the United Nations Millennium Development Goals (MDGs)^13^^,^^14^ and corresponding concerns about capabilities of African countries to achieve them, donor assistance, funding, and priority programs directed attention to the need for health systems strengthening in sub-Saharan Africa.^5^^,^^10^^-^^18^

Private funding, particularly from the Bill and Melinda Gates Foundation,^19^ has also been critical to the climate of support for health systems development. Global engagement with systems issues gained further traction in 2007, when an expert World Health Organization (WHO) panel developed a framework specifying essential components of health systems functioning comprised of 6 essential interdependent “building blocks.”^20^

This paper aims to take stock of this proliferation of financing, programming, and research that occurred in the wake of these historic milestones by conducting a bibliometric review of keywords associated with articles about health systems in sub-Saharan Africa published during the MDG era (1990–2014). Our aim is to clarify health systems themes emerging from the literature and gain insights that could address future research priorities and needs.

Several historic milestones prompted a climate of support for health systems development in the global health field.

## METHODS

We applied a bibliometric procedure to analyze scientific writing on health systems in sub-Saharan Africa published between 1990 and 2014. The term “bibliometrics” is characterized by its originators as “the application of mathematical and statistical methods to books and other media of communications.”^21^ (The term “scientometrics” is used interchangeably with the methods of bibliometrics.^22^) The assumption in conducting a thematic bibliometric analysis is that published literature directly reflects a body of scientific research and practice^23^ and that words that co-exist within abstracts and keyword lists represent ideational associations that can be used to interpret underlying themes and concepts. Termed “co-word analysis,” scales and associations can be constructed that reduce the volume of terms into indices representing common themes.^24^

Bibliometric analysis applies mathematical and statistical methods to terms used in publications to understand underlying themes and concepts.

### Data Preparation

This analysis reviews publications published between January 1, 1990 and June 6, 2014 and catalogued in the Elsevier Scopus database, one of the largest peer-reviewed citation libraries for science, technology, medicine, the arts, and humanities. We selected the Scopus database owing to its commitment to expert selection and assignment of indexed keywords to each article entry, including standardized Medical Subject Headings (MeSH) and Emtree medical terms (controlled vocabulary thesauri created by MEDLINE and Embase, respectively).^25^ Articles, reviews, notes, editorials, articles in press, and book chapters containing terms of interest that appeared in the title, abstracts, or keyword lists were selected, conditional upon the mention of sub-Saharan Africa as a region or inclusion of at least one sub-Saharan African country or subregion, allowing for linguistic or political contextual language commonly used.

For the purpose of this study, only index keywords selected by Scopus professional indexers were used, a procedure that avoids author-introduced indexing bias.^25^ Terms used to search for literature relevant to the analysis encompassed “health system,” “health care system,” “health program,” and “health service,” mindful of the fact that many activities undertaken by health programs have systems implications, but with the specific goal of reviewing literature that explains, describes, or guides the development of health systems in sub-Saharan African countries. Terms that conveyed no analytical meaning such as “human,” “article,” “male,” or “female,” as well as countries that are not part of the sub-Saharan African region, were eliminated. Only indexed keywords with an occurrence of 10 or more were selected for analysis. This preparatory process improved data quality, minimized ambiguity, and enhanced logical coherence of results.

Further analysis was conducted to compare articles published prior to the MDG era with those published during the MDG era. For this comparison, we used a stricter keyword occurrence cutoff of 25 or more and also applied relevance scoring to obtain higher specificity in the time analysis. Data were then divided by publication date to create 2 libraries of articles published during the pre- and post-2000 periods: 1990–1999 and 2000–2014, respectively. The final libraries were loaded into the bibliometric visualization mapping software VOSviewer,^26^ which calculates the optimal 2-dimensional scaling solution for the co-occurrence of the indexed keywords.

### The VOSviewer Co-Word Optimization Procedure

Co-word analysis assumes that underlying themes in a field of publication are defined by patterns of keywords that appear in lists provided by each publication.^27^ Thus, when a library of literature is analyzed, the ideas, concepts, and methods that constitute a field of knowledge are defined by clusters of keywords that reflect commonality within a field of scientific research.^21^^-^^27^ Although the notion that associations can be used to define underlying indices of relationships is not new,^28^ the application of these concepts to textual data is only recently gaining currency.^1^^,^^21^^-^^24^^,^^26^^,^^27^^,^^29^^-^^34^

Several alternative strategies for visualizing textual data are prevalent in the bibliometric literature.^30^^,^^31^ Some, such as the VOSviewer software, incorporate features that generate bibliometric maps for visualizing keyword associations in ways that are analogous to the astronomer’s depiction of objects in space:

- The frequency that terms appear defines the size of their visual representation as a labeled item.
- Their relative position in 2-dimensional space defines their relative association—all possible pairs of keywords that are commonly associated are positioned in close proximity of one another, and terms relatively unassociated with each other are mapped as remote from one another.
- Their clustering as sets of common colored keyword items define related conceptual domains, whereby sets of words that appear together more often in publications than can be attributed by chance share a common cluster of knowledge.

The relative position of keywords in a map is analogous to the concept of gravitation. The relative proximity of each possible pair is an index of the weight of their association, adjusting for all other possible associations in the keywords under study.

In practice, the operationalization of the mapping procedure applies optimization techniques to the positioning of groups of keywords in 2-dimensional space.^33^^,^^34^ The VOSviewer software, used for the analysis presented in this paper, modifies the standard optimization procedure for multidimensional scaling to maximize the common variance defined by the relational positioning of keywords.^34^ The VOS procedure also uses a relevance score to enhance the identification of ideational galaxies.^34^ The result of the computation process is a map in which terms that are relatively common to a general library of articles in an analysis appear at the center of a universe of knowledge, while keywords that are weakly associated are peripheral. By separating analyses by time of publication, clusters of terms defining galaxies can be created over successive time periods, allowing researchers to visualize changes in the thematic focus of research fields over time.

### Identifying Change Points in the Volume of Literature

In addition to the bibliometric analysis of keywords, we applied spline regression to test whether the publication volume trend changed over the MDG period relative to the trend in the 1990s. The term “spline regression” refers to an econometric method for testing hypotheses that a time series trajectory has changed at a discrete point in time. The method estimates the relative slope of trajectories and the point in time that a significant disjuncture occurred.^35^ To pursue this analysis, “changepoints” are statistically defined in the pace of health systems publications in sub-Saharan Africa during the period of 1990 to 2013. We excluded 2014 on the basis of incompleteness. Our motivation for pursuing this line of inquiry was to divide the literature into time groups representing citations published before and after these “changepoints” so that they may be thematically mapped using the VOSviewer software. These thematic maps, in turn, could clarify how the pace of health systems research changed over time in response to key events. To identify significant “changepoints,” we used spline regression models of the form:

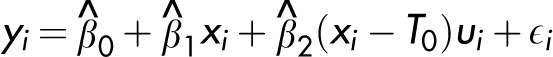



where,

*y_i_ = *The number of publications in year *i*
*x_i_ = *Integer year of publication tally
*T*_0_ = The potential changepoint year
*u_i_ = *A step function which is equal to 0 if *x_i_ < T*_0_ and is equal to 1 if *x_i_  =  T*_0_.
∊*_i_*  = An error term for year *i*

Spline knot values from 1991 to 2012 were estimated and compared using Akaike’s Information Criterion (AIC).^36^ The knot from the model with the lowest AIC was selected as the “changepoint.” We then applied splines of increasing polynomial order to optimize fit. This procedure permitted appraisal of whether the advent of the MDGs in 2000–2001 accelerated the pace of publication through inference on the models with knots at 2001. An estimate for *β*_2_ that is positive and significantly different than zero is indicative of an acceleration. This hypothesis was tested using t-tests and an *α*  =  .05 level of significance.

### Hypotheses

Hypotheses guiding this analysis are implied by keywords that are associated with global frameworks for cross-cutting issues in health systems research and policy. In particular, the WHO health systems building blocks define essential capabilities for sustaining and strengthening health systems functioning (Table).^20^ We posited that the health systems literature emerging from Africa would reflect this consensus, with clusters of keywords corresponding to the 6 WHO building blocks. Moreover, there are essential elements of successful health care functioning that define consensus thinking about the essential elements of successful primary health care in Africa. These include research on the quality of care, effective communication within systems and with the population served, effective organizational functioning, appropriate capabilities to use and scale-up innovation, means of adapting systems to social contexts, and strategies for maximizing access to essential care through scaling-up innovation or organizational reform (Table).^1^^,^^23^ Bibliometric maps are expected to visualize clustering, centrality, and ideational content that reflect these compelling appeals for “systems thinking.”^2^^-^^5^^,^^37^^-^^53^ We therefore posited that the 10 themes outlined in the Table would be reflected in bibliometric estimates of relationships comprising keywords in our database.

**TABLE:** Expected Keyword Themes From sub-Saharan African Health Systems Publications Published Between 1990 and 2014, Based on Global Frameworks for Health Systems Development

Health Systems Frameworks	Expected Keyword Themes	Related References
**WHO Building Blocks (World Health Organization^20^)**		
1. Access to essential health technologies	Expanding health coverage	Kruk^37^
	The range of health care options: the development, provision, and evaluation of health technologies and access to technologies	Travis et al.,^5^ Fonn^38^
	Community health services: community health centers, community health worker, community health planning, community participation, community engagement	Freeman et al.^39^
	Quality assurance, quality management, quality indicators, quality improvement	Kinney et al.^40^
2. Availability of providers of health services	Manpower and personnel operations: the training, deployment, and management of service providers	Cometto, Campbell, & Sheikh^41^
3. Information resources for health service decision making	Health information management systems: the design, implementation, and use of information for decision making at critical levels of the system	Boerma et al.^42^
	Communication and knowledge management: interdisciplinary communication, dissemination, research utilization, organizational communication	Shakarishvili et al.^43^
4. Capabilities to provide equipment, facilities, and supplies for operations	Logistics systems: the implementation, evaluation, or reform of logistics, equipment procurement, facilities development, and commodity supply systems	Bornbusch & Bates^44^
5. Planning, budgeting, and financing operations	Financial planning and management: activities for planning, budgeting, and managing resources for sustaining services	Friberg et al.^45^
6. Provision for leadership and governance of the health care system	Leadership systems: operations for developing, implementing, and sustaining leadership and governance systems	Fiszbein, Ringold, & Rogers^46^
**Cross-Cutting Research Themes**		
7. Systems research	Inter-building block themes, multilevel analysis, systems research, mixed qualitative and quantitative measurement, systems evaluation, operations research, implementation science	deSavigny & Adam^47^
	Experimental, quasi-experimental, and plausibility designs; evaluation methods	Remme et al.,^48^ Habicht et al.^49^
8. Organizational diagnosis	Bottlenecks, malaise, corruption, theft, mismanagement	Gilson & Mills^50^
**Cross-Cutting Implementation Themes**		
9. Scaling-up organizational change	Scaling-up, decentralization, using innovation, restructuring	Simmons et al.,^51^ Yamey^52^
10. Adaptive systems: “open systems” indicators	Social organizational context: economic status, educational attainment, gender issues, family characteristics, family relationships, social organization	Shalley & Gilson,^53^ Gilson et al.^3^

### Limitations

No bibliometric map is an exact representation of reality. Restricting the textual complexity of an entire body of scientific knowledge to size, color, and distance displayed across 2 dimensions will always be a simplification of the governing web of relationships portrayed. However, the bibliometric map represents an exploratory tool that can suggest associations, in analogy to the correlations that can be calculated in statistical analyses. Just as correlations are associations that are not necessarily causally defined, bibliometric associations and clusters are exploratory rather than explanatory.

The VOSviewer assumes that a web of co-occurrence can be adequately captured in 2 dimensions. In fact, multidimensional spatial analysis may be required for a given investigation, particularly if concepts and issues under investigation are too complex to define in the restrictive assumptions that the procedure employs.

A further limitation concerns mapping bias that could arise from our choice of a cutoff for keyword occurrence. Inclusion of all keywords contributes “noise” that arises from the tendency of authors or Scopus reviewers to list synonyms as separate keywords. We have attempted to eliminate obvious redundancy, but the inclusion of all keywords, no matter how rare their occurrence may be, obfuscates rather than clarifies the visualization process. We have determined that limiting the analysis to keywords that appear at least 25 times in the articles under review is consistent with the goal of analyzing nearly all of the articles in the library. Lower cutoff values would not add articles to the analysis.

Health systems development often involves donor-funded projects and consortia that produce manuals, conferences, reports, and web-based products that are unpublished an unindexed by Scopus or other citation databases. Published papers may inadequately represent the health systems contribution of unpublished reports.^54^ However, the focus of our review remains the products of scientific and policy teams and their peer-reviewed publications.

A final limitation concerns the challenge of representing the results of data visualization in publication-based maps. The number of keywords and their relationship is an intractable problem of graphics. To address this challenge, we present our results at http://arches.columbia.edu/health-systems-research/ in a format that permits viewers to explore results, expand displays, and examine relationships within clusters with the same degree of flexibility that the authors have been equipped by VOSviewer to pursue.

## RESULTS

**FIGURE 1:**
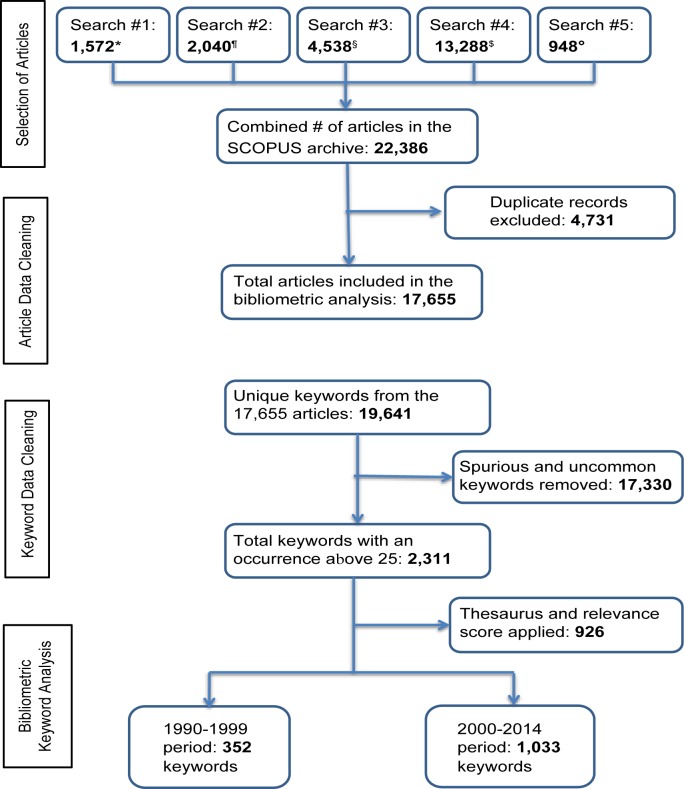
Scopus Search Strategy and Bibliometric Data Preparation Process * TITLE-ABS-KEY (“health system”) + full country list ^¶^ TITLE-ABS-KEY (“health care system”) + full country list ^§^ TITLE-ABS-KEY (“health program”) + full country list ^$^ TITLE-ABS-KEY (“health service”) + full country list ° TITLE-ABS-KEY (“health system” or “health care system” or “health program” or “health service”) + (all African Regions)

Our search initially yielded 22,386 articles. After excluding duplicate records, 17,655 articles remained, which comprised the final set of articles included in the bibliometric analysis (Figure 1). From these articles, 2,240 keywords occurring at least 10 times were included in the overall analysis of the literature published between 1990 and 2014, and 2,311 keywords occurring at least 25 times were included in the 2 time analyses comparing the pre-MDG era with the MDG era. After applying thesaurus and relevance scoring to the 2,311 keywords,^34^ 352 keywords from the 1990–1999 period remained and 1,033 keywords from the 2000–2014 period.

**FIGURE 2:**
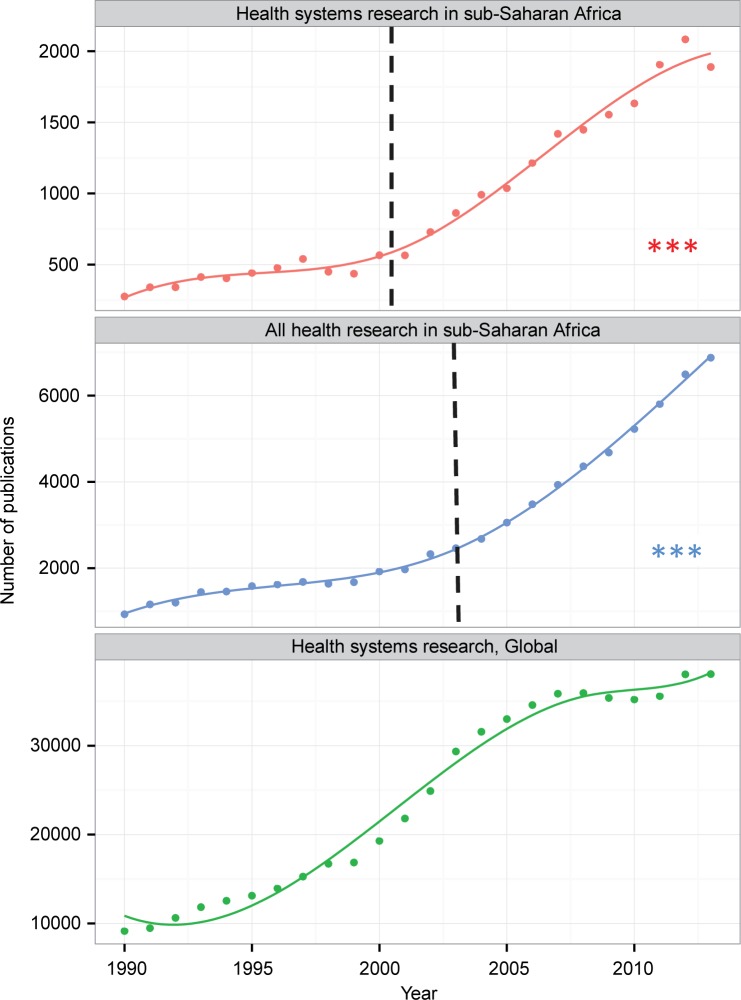
Annual Volume of Publications About sub-Saharan African Health Systems Research, sub-Saharan African Health Research in General, and Health Systems Research Globally, 1990–2014 The point at which the volume of literature changed, estimated using spline regression, is shown with a dotted line, which occurred at the advent of the Millennium Development Goals (MDGs) in 2000–2001 for health systems research in Africa and in 2003 for health research in Africa. Statistical significance at *P* < .0001 is denoted with 3 asterisks. There was no association of the onset of the MDG era with global health systems research.

As Figure 2 shows, the volume of health systems publications expanded over the 1990 to 2014 period both globally and for the sub-Saharan African region. (The data for literature on health research in sub-Saharan Africa and for literature on health systems research globally are based on trends in much larger datasets than the main dataset of 17,655 articles analyzed in this paper, which focuses specifically on health systems research in sub-Saharan Africa.) The rate of increase in this expansion accelerated significantly in 2003 for all health research in Africa and in 2001 specifically for health systems research from the region. There is no evidence of an MDG-associated upward trend for health systems research globally. However, the overall rate of global expansion of publication was more pronounced than was observed in sub-Saharan Africa. Nonetheless, the expansion of health systems publications over the period was substantial in Africa, with regression results suggesting an acceleration in the immediate post-Millennium era. In 2000, only 460 sub-Saharan African health systems articles were published with keywords connoting a focus on health systems topics; by the end of 2013, this annual figure had increased to 1,401.

The volume of publications on health systems in sub-Saharan Africa expanded between 1990 and 2014, from 460 articles in 2000 to 1,401 by the end of 2013.

### Themes in the sub-Saharan African Health Systems Literature, 1990–2014

**FIGURE 3.**
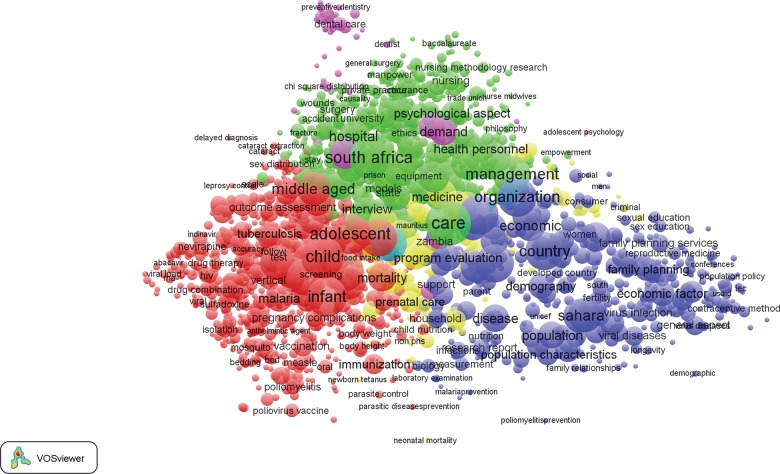
Bibliometric Map of 2,240 Keywords From 17,655 Publications on Health Systems in sub-Saharan Africa, 1990 to 2014 The relative size of each circle corresponds to the keyword occurrence among the 17,655 publications. The circles are, in turn, grouped into thematic clusters represented by a common color: green, personnel (mainly nursing) issues; purple, dental health; blue, family planning programming; yellow, maternal health; and red, indicators of morbidity and mortality.

Figure 3 presents a visualization of bibliometric results for the 17,655 publications published between 1990 and 2014 that are included in the analysis. In this map (and subsequent maps included in this article), the relative size of each circle corresponds to the keyword occurrence in our health systems library; the circles are, in turn, grouped into thematic clusters represented by a common color.

Clustering results are inconsistent with our posited configuration of keyword galaxies. Rather than reflecting systems themes, bibliometric galaxies define 5 domains of health science research and action:

1. A red cluster that is dominated by indicators of morbidity, mortality, and relevant research themes
2. A blue cluster dominated by indicators of family planning programming and related outcome indicators and social and behavioral determinants
3. A green galaxy for systems indicators that focus on research, training, and policies concerning personnel issues, mainly regarding nursing
4. A cluster shaded yellow at the center of Figure 3 defining a thematic focus on maternal health
5. A small and peripheral purple cluster identifying dental health and related topics

This configuration of themes contrasts with health systems frameworks and relationships anticipated in the Table.^3^^,^^37^^-^^53^ For example, keywords related to the WHO building block of “access to essential health technologies” are dispersed in Figure 3. Topics that define systems planning are equivalently dispersed: “organization,” “health care delivery,” and “health care planning” appear in the blue cluster, while health care personnel indicators are in the green cluster, and keywords for “program” appear in the red cluster.

Clustering of keyword themes from the published literature on health systems in sub-Saharan Africa do not coincide with the 6 WHO building blocks.

**FIGURE 4:**
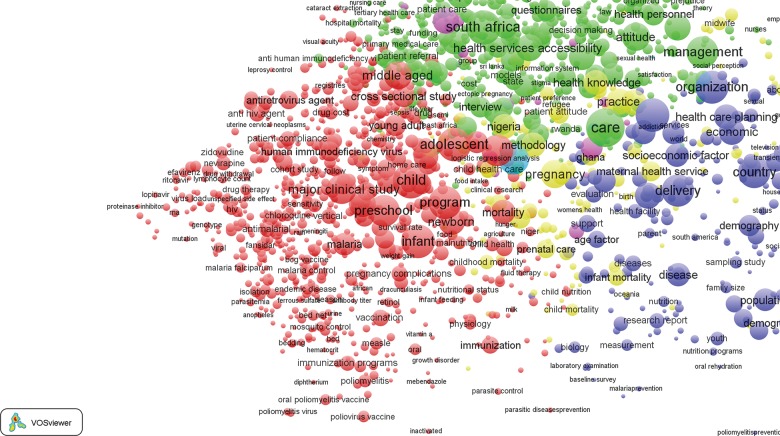
Bibliometric Map of Keywords From Publications on Health Systems in sub-Saharan Africa, 1990 to 2014: Expansion of the Red Cluster of Keywords Related to Morbidity and Mortality Indicators

Despite this counter-systemic clustering, there is evidence of coherent clustering of health research themes in general. Zooming in on specific sections of Figure 3 provides clarification of the labeling of terms in each major cluster. For example, keywords most central to Figure 3 (i.e., the red cluster) and intensely cited in the literature concern services for primary health care patients,^55^ organizational issues related to provider-client relationships,^55^^-^^57^ access to care,^37^^-^^40^^,^^55^^-^^57^ the quality of services,^50^^,^^56^^,^^58^^,^^59^ and the management of frontline workers^60^ (Figure 4). These “high-density” keywords comprise indicators of the provision of essential care or indicators of demand for care and primary health care service themes that are deemed crucial to accelerating the MDGs.^5^^,^^17^^,^^18^^,^^40^^,^^47^^,^^60^^,^^61^ Keywords relevant to quality of care cluster with indicators of epidemiological research on morbidity and mortality (e.g., malaria, fever, tuberculosis, or conditions associated with pregnancy) or covariates defining age categories,^40^^,^^55^^-^^58^^,^^62^ such as adolescents, infants, or children under 5, as shown by the red cluster in Figure 3 and the zoomed-in version of Figure 4. Indicators of epidemiological methodology or experimental designs or covariates concerning indicators of social, familial, or behavioral characteristics of epidemiological research appear in the red cluster.

**FIGURE 5.**
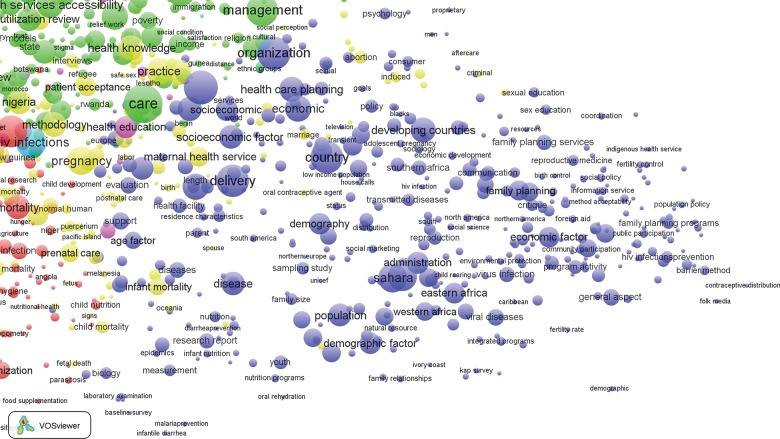
Bibliometric Map of Keywords From Publications on Health Systems in sub-Saharan Africa, 1990 to 2014: Expansion of the Blue Cluster of Keywords Related to Family Planning Programming

Indicators of the promotion, delivery, and evaluation of reproductive health and family planning programs appear as the blue cluster in Figure 3, which is expanded in Figure 5. Demographic research and reproductive health topics appear in this cluster, together with covariates of the determinants of reproductive behavior, such as household economic status. Organizational issues concerning the delivery of care, planning, and service delivery models cluster with reproductive health indicators.

**FIGURE 6:**
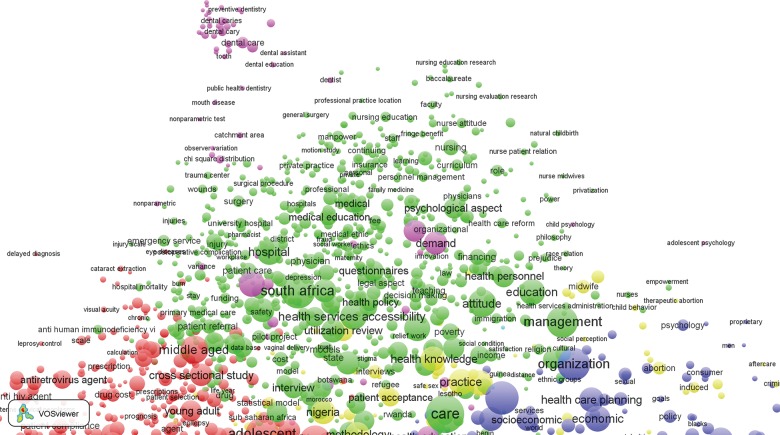
Bibliometric Map of Keywords From Publications on Health Systems in sub-Saharan Africa, 1990 to 2014: Expansion of the Green Cluster of Keywords Related to Personnel Issues

The “green cluster,” expanded in Figure 6, has multiple indicators representing management, training, and deployment of personnel. High-density keywords in this cluster define the location of research, most prominently South Africa, where systems research is more common than in any other country. Other countries where HIV research is well developed, such as Kenya, also appear in the green cluster. But South Africa is the dominant country represented in this literature, with articles and activities that apply to all domains of health systems research but most prominently represented by research on access, utilization, manpower issues, health insurance, and other organizational issues. Personnel management, service utilization, indicators of care systems, and financing appear in this cluster. Although topics are peripheral in Figure 6, most indicators of manpower and training and manpower development concern nurse training, nurse deployment, and related frontline worker capacity-building themes rather than manpower development themes related to systems strenghening.^18^^,^^38^^,^^63^

Publications about other manpower issues concerning supervisory or management training are relatively uncommon. Similarly, the other WHO building blocks concerning information for decision making, essential commodity supply/logistics systems, and planning and budgeting are uncommon, although considerable attention is directed to costing analyses and health economics research. Somewhat surprisingly, keywords related to the building block concerning leadership and governance systems and organizational safeguards are uncommon in the literature, apart from a limited research on community engagement.

**FIGURE 7:**
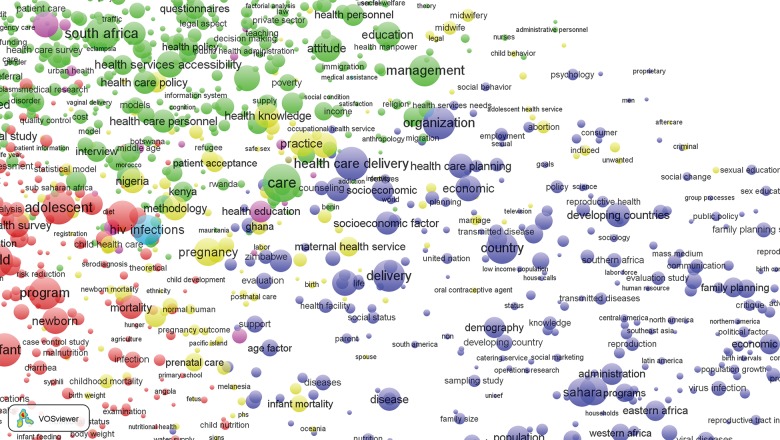
Bibliometric Map of Keywords From Publications on Health Systems in sub-Saharan Africa, 1990 to 2014: Expansion of the Yellow Cluster of Keywords Related to Maternal Health

Maternal and newborn health determinants and assessment cluster at the center of Figure 3 in yellow, which is expanded in Figure 7. Topics concerning pregnancy, delivery, perinatal health problems, and prenatal and postnatal care cluster with abortion, unwanted fertility, and other indicators of the consequences of reproductive health problems. Infant and childhood mortality cluster with pregnancy outcomes.

#### Correspondence of Bibliometric Maps With the WHO Health Systems Framework

Prominent keywords from the WHO building blocks include those concerning access to care (the first building block) and related research on safety, efficacy, or service provision. Other themes falling within the WHO health systems strengthening framework are less common, peripheral, or weakly linked to other systems indicators.^5^^,^^37^^,^^40^ For example, although the WHO health system building block related to availability of human resources for health is represented in Figure 6 by keywords that connote nurse training, deployment, and management, there is less focus on more general systems themes of supervision, management, and systems capabilities that enable such workers to function effectively.^18^^,^^41^^,^^46^^,^^63^ Keywords connoting WHO building block themes concerning the design, implementation, and use of information for decision making,^18^^,^^63^^-^^67^ health information management, and research utilization are advocated in reviews, but as yet, are not prominent in the African health systems literature,^5^^,^^53^^,^^68^^,^^69^ and keywords concerning internal organizational communication and knowledge management^53^^,^^68^^,^^69^ are rare.^67^ Keywords from literature focused on the building block of implementation, evaluation, or reform of logistics, equipment procurement, facilities development, and commodity supply systems^44^^,^^70^ are also rarely pursued. The fifth WHO health systems building block represents planning, finance, budgeting, and managing resources for sustaining services.^45^^,^^71^ Health economics research is prominent in the health systems literature, but keywords relevant to the measurement of the strength, readiness, and functioning of health systems^72^^,^^73^ are not, suggesting that management and systems science is making less of a contribution to sub-Saharan African health systems publications than other health science or general policy topics. Clearly, apart from extensive work on the (first) care and access building block, the informative and widely cited WHO framework has yet to demonstrate bibliometric evidence of an impact on country-specific health systems writing and research.

The only WHO building block that is prominently represented in the published literature on health systems in sub-Saharan Africa is that of access to health care.

This conclusion is particularly salient for the sixth “governance” building keywords that connote indicators of the implementation and evaluation of strategies for developing, managing and sustaining leadership.^74^^-^^79^ Results show that keywords associated with this theme are uncommon. One notably peripheral keyword display is labeled “corruption,” but indicators of organizational diagnosis, bottlenecks, mismanagement, dysfunctions, or malaise ^80^ are so rare that relevant terms do not appear in the bibliometric maps.

#### Correspondence of Bibliometric Maps With Cross-Cutting Research and Implementation Themes

International reviews have emphasized the importance of systemic research cutting across multiple WHO building blocks denoted by keywords from the fields of systems analyses,^45^^-^^47,50^ multilevel analyses,^40^^,^^47^ recursive processes,^5^^,^^18^^,^^47^^,^^68^^-^^71^^,^^81^^-^^85^ and implementation science or research.^85^^-^^90^ Yet keywords connoting research methodologies and designs for complex systems research, as advocated in widely disseminated reviews,^2^^,^^3^^,^^5^^,^^47^^,^^48^^,^^50^ do not yet generate keyword identifiers. Instead, country-specific studies refer to the methodologies of epidemiological or socio-medical research rather than to the application of systems research methods such as organizational diffusion studies,^91^ systems trials,^82^^,^^90^ plausibility studies,^49^ implementation research,^87^^,^^88^ or topics concerning processes that are essential to understanding the systems requirements of organizational sustainability, resilience in times of crisis,^92^ or restructuring and reform.^3^^,^^18^^,^^43^^,^^53^^,^^55^^,^^68^^,^^69^

Implementation science and other research that cuts across the WHO building blocks do not appear in the published literature on sub-Saharan African health systems.

In addition, the determinants of systems change^93^ and of scaling-up innovation^51^^,^^52^^,^^93^ are reviewed and advocated in the literature,^51^^,^^52^ but keywords connoting evidence-based scale-up of program innovation appear in the form of reviews of what is needed rather than keywords portraying actual work on the ground.^93^ The terms organization, management, and administration are common in the literature, but keywords that are frequently used in the commercial and business literature for using and scaling-up systems innovations are uncommon in the sub-Saharan African health systems literature. Keywords rarely connote the application of processes of systems strengthening, evidence-driven reform and restructuring, or translating operations research into action. Apart from frequent reference to the need to scale-up innovation, results suggest that country-focused progress with monitoring of the pace, content, and fidelity of scaling-up processes have yet to be noted in the health systems literature. While scaling-up is a prominent theme in the health literature, application of this concept applies mainly to the use of discrete research findings or to the introduction of new clinical modalities rather than to processes of scaling-up systems development.

Keywords related to scale-up appear in the form of what is needed rather than the process of scaling-up.

When terms connoting delivery and care appear in Figures 3–7, their reference is mainly to public-sector programs rather than to the role of the private sector in African health systems. Although some themes concerning privatization, commercial outlets, and social marketing are evident, their position in maps is peripheral to the general configuration of Figure 4, Figure 5, Figure 6, and Figure 7.

The conclusion that emerges from our expansions of Figure 3 is a lack of systematic attention to organizational process research and social organizational contexts of health systems, such as open systems research and terms relevant to the adaptive social development of systems of care, as advocated by Shalley and Gilson (2004),^53^ and others. Contextual considerations in the design of systems, connoting open systems indicators of community participation, social organization, or other contextual factors, are notably peripheral or associated primarily with efforts to improve the functioning of family planning programs. Population and demographic keywords in Figure 5 are isolated from health manpower terms in Figure 6 and from maternal health systems indicators in Figure 7. Demographic research and population-based trials are focused on family planning topics; experimental or quasi-experimental systems intervention trials are uncommon. Indeed, terminology related to systems development plausibility trials and study designs that test policy options rarely occur. Evidence based on complex systems interventions that cut across domains of the WHO building blocks and test the impact of health systems strengthening on health, survival, or fertility is rarer still.^4^^,^^90^ Examples of exceptions to this generalization are health systems development trials that have been conducted in Mozambique,^94^ Zambia,^95^ Rwanda,^96^ Tanzania,^97^ and Ghana.^98^ Although new initiatives have been launched in response to the general gap, with the goal of generating interdisciplinary science focused on health systems development,^90^ complex systems trials of reform packages remain rare.

Taken as a representation of health systems research, the topics displayed in bibliometric maps presented in this article are more appropriately characterized as epidemiological studies with service indicators as covariates rather than as systems analyses of interlocking components of organizational functioning. Terms connoting processes that determine the effective scale-up of experiments, the utilization of results, or evidence-based policy are uncommon and unassociated with study designs. Endpoints for research are morbidity, mortality, or fertility indicators rather than systems variables, implementation indicators, or organizational functioning. Quite surprisingly, implementation science is as of yet a “black hole” in the astronomy of health systems research in Africa.

### The Health Systems Literature Before the MDGs, 1990–1999

**FIGURE 8:**
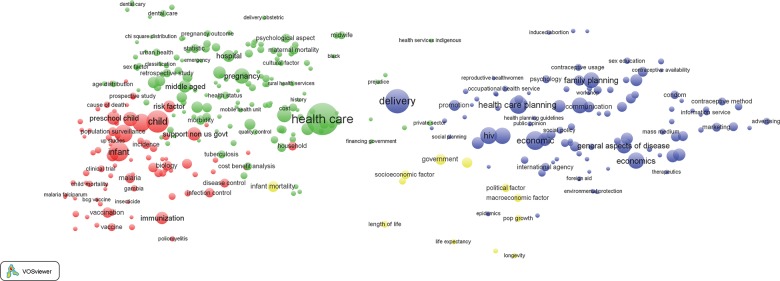
Bibliometric Map of 352 Keywords From Publications on Health Systems in sub-Saharan Africa, 1990 to 1999 Thematic clusters are represented by a common color: red, health technological research; green, maternal and oral health services; yellow, health policy and life expectancy; and blue, health care delivery, economic and social covariates of health, and family planning.

The map in Figure 8 portrays thematic simplicity of the health systems literature of the pre-MDG era. Only 4 keyword clusters represent the library’s underlying themes:

1. Health technological research with demographic and infectious disease morbidity determinants, prevention, and outcomes (red cluster)
2. Health care services related to pregnancy, maternal health, oral health and social covariates (green cluster)
3. Health policy and life expectancy keywords (yellow cluster)
4. Health care delivery and planning, economic and social covariates of health, and family planning (blue cluster)

This ideational simplicity is combined with a polarization of the map into separate domains for health (left side, Figure 8) and program design and family planning (right side), a configuration of literature that is consistent with the overarching importance of vertical programming in sub-Saharan Africa over the 1990 to 1999 period. Keywords such as delivery, health care, and government, with 891, 1,542, and 183 occurrences, respectively, form the most central keywords. At the center of the diagram, the keyword “delivery” in blue and “health care” in green, suggest that indicators of the provision of health services were central to the general body of literature, with the term “health care” clustering with terms of fixed-facility functioning and delivery (green cluster) as well as family planning indicators and social determinants of reproductive health (blue cluster). But the dominant keyword is “delivery” rather than indicators of how delivery systems are developed, changed, or strengthened.

Before the MDGs, provision of health services was central to the general body of literature on sub-Saharan African health systems.

Themes in red connoting morbidity outcomes, such as malaria and tuberculosis, and infant and child health and survival endpoints, cluster with green health care and maternal health themes on the left side of Figure 8. This cluster is remote from the literature galaxy in blue—the cluster of keywords related to health delivery, family planning, HIV, and financing. The lack of central cross-cutting keywords or clusters connotes a distinct lack of integration between health from an epidemiological perspective (red and green clusters) versus health systems thinking in the 1990s. The yellow cluster representative of government and life expectancy is relatively central but lacks the degree of density that would connote thematic cohesion.

### The Health Systems Literature After the MDGs, 2000–2014

**FIGURE 9:**
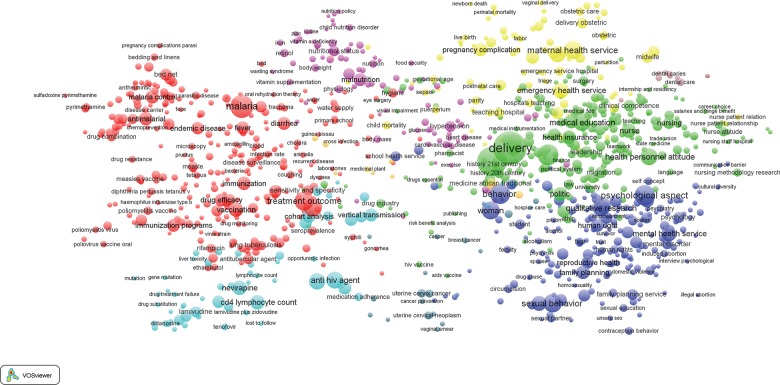
Bibliometric Map of 1,033 Keywords From Publications on Health Systems in sub-Saharan Africa, 2000 to 2014 Thematic clusters are represented by a common color: red, health technological research; green, manpower and training; yellow, maternal and oral health services; blue, health care delivery, economic and social covariates of health, and family planning; purple, non-communicable diseases; turquoise, HIV/AIDS; and a small peripheral cluster at the top right for dental public health.

The configuration of Figure 9 for the post-2000 era represents a continuing prominence of service delivery, but a shift in the focus of health systems publications after 2000. Seven key clusters emerge:

1. A red cluster, with a focus on infectious disease morbidity as in Figure 3, except for the separate clustering for nutrition and non-communicable disease indicators and for HIV/AIDS keywords
2. The nutrition and non-communicable disease indicators are shaded purple
3. The new turquoise cluster represents HIV/AIDS keywords
4. The yellow cluster for maternal health keywords remains as a separate cluster but is shifted to a peripheral position, indicating diminished centrality
5. A blue cluster is associated with family planning and reproductive health as well as reproductive tract cancers as in Figure 3 and Figure 8, but with more evidence of keywords on gender and social issues
6. A green cluster, as in Figure 3 and Figure 8, but with less emphasis on particular countries such as South Africa, and more general themes concerning manpower, leadership, and other systems issues
7. A peripheral and minor cluster at the top right for keywords associated with dental public health

The red cluster and the emergence of the separate turquoise cluster parallel the funding pattern of prominent international institutions and foundations such as The Global Fund to Fight AIDS, Tuberculosis and Malaria and the Bill and Melinda Gates Foundation, with a preponderance of keywords related to programs and technologies for the treatment and prevention of HIV/AIDS and malaria, tuberculosis, respiratory infections, and other diseases of childhood, as well as prevention programs such as immunization promotion or polio eradication. Although thematic clusters related to medical modalities and morbidity outcomes (left side of Figure 9) remain separate from clusters denoting health systems (right side of Figure 9), the overall density and centrality of the map is suggestive of higher integration of the health systems literature in general, a possible consequence of thematic integration and MDG focus.

Although medical modalities remain separate from health systems in MDG-era publications, there is a general trend of greater integration of the concepts than in the 1990s.

In comparison to Figure 8 (pre-MDG), cross-cutting themes represented by Figure 9 are more numerous and emerge from different clusters that bridge the gap between epidemiological and systems keyword indicators. The purple cluster with terms such as malnutrition, obesity, and hypertension reveal an emerging focus on non-communicable diseases. Furthermore, the association of this cluster with non-communicable diseases portrays an emerging focus on sources of the burden of disease that were not priority themes of the Alma Ata “Health for All” agenda.^9^ The continuing focus on infectious disease, in conjunction with the emerging focus on the management and prevention of non-communicable disease, is consistent with the development of a health systems literature that has an increasingly balanced focus on the burden of disease, in general, rather than a literature that is focused on childhood illness and infectious disease.

The map in Figure 9 is also indicative of greater thematic diversity after 2000 than what prevailed in the 1990s: reproductive health and maternal health keywords remain prominent in the post-Millennium era but are arrayed as equidistant clusters from the center of the map, suggestive of greater integration. Health systems indicators (shaded green) are more prominent, more often indicators of general policy themes, and coterminous with service delivery indicators. This feature of Figure 9 is representative of the growing consistency of the health systems literature with systems thinking itself. In particular, keywords associated with the green cluster mirror the 6 building blocks of the WHO Health Systems Framework^20^ and include keywords describing policy, service delivery, education, personnel and personnel development, and financing. The proximity of these keywords with other cross-cutting themes such as family planning, reproductive health, and emergency care further support the notion of the emergence of a more coherent health systems literature in the 2000 to 2014 period than had prevailed in the 1990s.

While the comparison of Figure 8 and Figure 9 is suggestive of greater integration, the literature throughout both periods portrayed is more focused on epidemiology, socio-demographic, and health research rather than systems analysis per se. Indeed, the dominant new theme in the health systems literature in the post-Millennium era is HIV/AIDS. An entire HIV cluster (turquoise) emerges in the post-2000 period that is separate from other sources of morbidity. Keywords related to HIV/AIDS in the pre-2000 bibliometric map of Figure 8 (blue cluster) represent HIV/AIDS strictly from an infectious disease standpoint. Concomitance with keywords such as family planning and sexual behavior further exemplifies how HIV was perceived as contributing to sexually transmitted infections rather than a standalone pandemic. The post-Millennium map of Figure 9 is indicative of thematic change with HIV/AIDS becoming a prominent theme of health systems research, possibly reflecting the impact of the US President’s Emergency Plan for AIDS Relief (PEPFAR) and the Global Fund, as well as the establishment of MDG Number 6 to combat HIV/AIDS, malaria, and other diseases. In Figure 8, HIV/AIDS is represented by 4 keywords and 1,144 occurrences, whereas in Figure 9, HIV/AIDS has a distinct cluster comprised of 59 keywords and 7,375 occurrences.

A new HIV/AIDS cluster emerges in the post-2000 publication era.

Thematic shifts from Figure 8 that are evident in Figure 9 attest to the well-known influence of international investment in research, implementation, and policy trends and themes in sub-Saharan Africa. From the onset of post-independence development of health service implementation, capabilities and subsequent investment in the implementation of modality and disease-control focused health initiatives, health systems revenue, capacity, and dissemination capabilities have developed markedly throughout the region. For example, systems policies, plans, and action in Africa were influenced by the “Expanded Programme for Immunizations,”^99^ the WHO-sponsored “Integrated Management of Childhood Illness” initiative,^100^ and global programs focused on the control of specific diseases or the amelioration of health problems. Initiatives fostering system integration, reform, and decentralization directed particular attention to Africa.^101^ World Bank programs in sub-Saharan Africa prior to the MDG area were also influential.^10^ Even initiatives that were not directly systems focused, such as the smallpox eradication campaign, the global campaign against malaria, and the polio eradication campaign, contributed to systems development.^6^ The 1978 World Health Assembly was transformational,^8^ and the challenge of implementing its primary health care agenda was productive.^9^

Family planning programs developed in the early African post-independence era^102^ were expanded in the pre-MDG era, first with US support commencing in the 1960s^103^ and subsequently by the emergence of support from the United Nations Population Fund (UNFPA) and World Bank.^104^ Response to this investment in the health systems literature has been catalyzed, in part, by international conferences^103^ and by expanding commitments of European and foundation donors to service system development.^104^^,^^105^ The emergence of South Africa from apartheid and opportunities for its universities to engage in collaborative partnerships contributed significantly to health systems research in the region. Taken together, these initiatives underpin the health systems literature prior to 2000.

The MDG era was associated with an expansion of systems research and greater thematic diversity, reflecting greater attention to systems thinking within each domain of health research and policy dissemination.^5^^,^^10^^-^^18^^,^^68^^,^^69^ Bilateral and multilateral foreign aid initiatives associated with the MDG agenda provided additional impetus for health systems development.^2^^,^^12^^-^^15^^,^^17^^,^^104^ The expansion of foundation support, targeted on MDG goals, has been critical; support from the Bill and Melinda Gates Foundation for health initiatives has been transformative.^19^ In particular, the global response to the HIV/AIDS crisis generated revenue, research, and systems capability throughout Africa.^106^ International conventions, donor programs, and international priorities that emerged have accelerated investment in health systems more generally,^73^^,^^74^^,^^106^ enhancing capabilities to understand problems, document responses, and finance the pursuit of effective results.

## CONCLUSION

The volume of the health systems literature from sub-Saharan Africa has been expanding, with considerable potential for contributing to evidence-based systems development throughout the region. Critically important frameworks for action, research, and policy have been published and widely cited.^5^^,^^18^^,^^20^^,^^47^^,^^50^^,^^72^^-^^74^^,^^81^^,^^82^^,^^106^ The pace of expansion was associated with the onset of the MDG era, with concomitant acceleration in the publication and proliferation of themes consistent with systems deliberations. This expansion of analytical writing is much needed, as systems constraints to health development are widely acknowledged to be more prominent in sub-Saharan Africa than in any other region. The MDG era has been associated with shifts in the focus of health systems publication from polarized separation of family planning from primary health care into a more holistic body of literature reflecting a greater degree of integration. This conclusion is suggested by the contrasting configuration of pre-2000 literature from post-2000 publications.

Yet, despite this marked expansion of systems publication, the literature reviewed in this bibliometric analysis is expanding at a slower pace than is evident globally. Moreover, the content of country-specific systems writing, as portrayed by keyword galaxies, remains more focused on describing component solutions or disease-specific epidemiological outcomes of discrete technical interventions rather than on methods or results that focus on the process of changing organizational functioning or strengthening health systems. Bibliometric supernova that have emerged are indicators of specific diseases, such as HIV/AIDS or systems research in specific countries, such as South Africa. Keywords connoting “stars” in the constellation of knowledge are dispersed rather than grouped into prominent displays of health systems galaxies. There is an absence of thematic clustering that our health systems literature review was posited to portray.

Indeed, “systems thinking,” as promoted by several landmark reviews, commentaries, and frameworks and summarized in the Table, has yet to become a feature of Africa’s country-specific health systems literature. Results of our review lend support to recent appeals for greater attention to capacity building in health systems research and health systems trials,^90^ as well as greater attention to systems thinking in international donorsupported programs on the ground.^106^ Our hypothesis that bibliometric analysis would visualize elements of systems frameworks has not been supported by the results. Gaps are evident. There is a remarkable lack of attention to organizational malaise, dysfunction, corruption, or inefficiency. There is also a lack of attention to developing and deploying analytical and evaluation methods that are characteristic of holistic systems strengthening, a core goal of the initial MDG framework. Instead, the literature cites systems keywords as indicators in health research rather than as outcomes or endpoints in systems scientific investigations. Implementation science is underemphasized. If health systems are to be strengthened through the improved functioning, structure, and design of organizations that implement programs, then the challenge of designing, launching, and sustaining evidence-based organizational change and development must be a more concerted focus of health systems research in Africa in the future than has been the case in the past.

Systems thinking has yet to become a feature of Africa’s health systems literature.

## Notes

**Peer Reviewed**
